# Management Strategies for Posttransplant Diabetes Mellitus after Heart Transplantation: A Review

**DOI:** 10.1155/2018/1025893

**Published:** 2018-01-29

**Authors:** Matthew G. Cehic, Nishant Nundall, Jerry R. Greenfield, Peter S. Macdonald

**Affiliations:** ^1^Faculty of Medicine, St Vincent's Clinical School, University of New South Wales, Sydney, NSW, Australia; ^2^Heart Failure and Transplant Unit, St Vincent's Hospital, Sydney, NSW, Australia; ^3^Victor Chang Cardiac Research Institute, Sydney, NSW, Australia; ^4^Department of Endocrinology and Diabetes, St Vincent's Hospital, Sydney, NSW, Australia; ^5^Diabetes and Metabolism Research Program, Garvan Institute of Medical Research, Sydney, NSW, Australia

## Abstract

Posttransplant diabetes mellitus (PTDM) is a well-recognized complication of heart transplantation and is associated with increased morbidity and mortality. Previous studies have yielded wide ranging estimates in the incidence of PTDM due in part to variable definitions applied. In addition, there is a limited published data on the management of PTDM after heart transplantation and a paucity of studies examining the effects of newer classes of hypoglycaemic drug therapies. In this review, we discuss the role of established glucose-lowering therapies and the rationale and emerging clinical evidence that supports the role of incretin-based therapies (glucagon like peptide- (GLP-) 1 agonists and dipeptidyl peptidase- (DPP-) 4 inhibitors) and sodium-glucose cotransporter 2 (SGLT2) inhibitors in the management of PTDM after heart transplantation. Recently published Consensus Guidelines for the diagnosis of PTDM will hopefully lead to more consistent approaches to the diagnosis of PTDM and provide a platform for the larger-scale multicentre trials that will be needed to determine the role of these newer therapies in the management of PTDM.

## 1. Introduction

Diabetes mellitus is a common complication after heart transplantation. In the most recent report of the International Society of Heart and Lung Transplantation (ISHLT) Registry, the prevalence of diabetes mellitus was 23% at one year increasing to 37% at 5 years after heart transplant [1]. Posttransplant diabetes mellitus (PTDM) has been associated with increased rates of serious infection [2, 3], graft-related complications such as graft rejection and graft loss [4], and reduced long-term survival compared to nondiabetic recipients [1]. Consequently, The International Society of Heart and Lung Transplantation has recommended that routine screening for PTDM be performed with appropriate protocols in place for subsequent treatment [5]. The majority of studies that have examined treatment of PTDM have been conducted in renal transplant recipients; however management strategies for PTDM after renal transplantation may not be appropriate for heart transplant recipients. Heart and renal transplant recipients are both prone to high rates of renal dysfunction over time (mainly related to long-term calcineurin inhibitor use). However the risk of urinary tract infection is much higher after kidney transplantation [6], which may have implications for the tolerability and safety of SGLT2 inhibitors in renal transplant recipients. In addition, whilst the incidence of PTDM after kidney transplantation appears to be declining [7], the incidence after heart transplantation has been increasing steadily with the reported prevalence of PTDM at 5 years after transplant increasing from 32% in 2002 [8] to 37% in 2016 [1].

Long-term survival following heart transplantation has improved significantly in the modern era, largely due to the more proficient immunosuppressive regiments now available [8]. However, the diabetogenic effects of these immunosuppressive agents have contributed to increased rates of PTDM [4]. Various management strategies exist for controlling diabetes amongst the general population. However, no specific protocols have been created for managing PTDM following heart transplantation. There exists a significant need for prospective trials in this area, as PTDM continues to become an increasingly important issue in the transplant setting.

## 2. Definition of Posttransplant Diabetes Mellitus

New onset diabetes after transplantation (NODAT) has been recognized as a complication of solid-organ transplantation for over 50 years [9]. However, prior to 2003, when the International Consensus Guidelines on New Onset Diabetes after Transplant [10] were adopted, there was a lack of a standardized definition for NODAT. The term was defined as a “heterogeneous condition of abnormal glucose tolerance with variable onset, duration and severity” [10]. The most recent recommendation from an international consensus meeting held in 2013 [11] was that the term “Posttransplant Diabetes Mellitus” (PTDM) replaces NODAT due to a high prevalence of undiagnosed pretransplant diabetes mellitus. PTDM is defined as “newly diagnosed diabetes mellitus (DM) in the posttransplant setting (irrespective of timing or whether it was present but undetected prior to transplantation or not)” [11]. The reasoning behind this recommendation was that patients on the waiting list for transplantation are not routinely evaluated for the presence of diabetes mellitus using standard diagnostic methods such as oral glucose tolerance testing. Consequently, the diagnosis of diabetes after transplantation cannot accurately be described as new onset diabetes if no attempt was made to establish whether it was present prior to transplant.

Earlier studies of PTDM in heart transplant recipients reported incidence rates between 13 and 33% across various studies [12–15]; however no studies have been published since the updated criteria were released in 2014, which exclude hyperglycemia occurring in the immediate posttransplant hospitalization and follow-up.

The 2003 guidelines based the diagnostic criteria for posttransplant diabetes on the currently accepted definition for diabetes mellitus (Box 1), as defined at the time by American Diabetes Association (ADA) and World Health Organization (WHO) [16, 17] for nontransplant patients. The second international consensus meeting on PTDM used the 2003 guidelines as its foundation. The expert committee reevaluated the addition of hemoglobin A1c (HbA1c) as a diagnostic criterion, as it had been defined by ADA in 2010 for assessing diabetes status in nontransplant adults. It was concluded that HbA1c could be used to diagnose diabetes if elevated (≥6.5%); however, it should not be used alone to diagnose PTDM, especially in the first year following transplantation, as a normal HbA1c does not exclude the diagnosis in the presence of posttransplant anaemia or renal dysfunction [11]. HbA1c readings of 5.7–6.4% need to be followed up with the aforementioned testing methods; however, an HbA1c > 6.5% is unlikely to be a false positive [11].

Whilst PTDM encompasses all recipients with diabetes following transplantation, there is an increasing number of patients with pretransplant diabetes mellitus (Pre-Tx DM) as diabetes no longer remains an absolute contraindication to transplantation [26]. Individuals with diabetes, but without secondary end-organ damage (proliferative retinopathy, nephropathy, or neuropathy), have achieved excellent long-term outcomes [27]. However patients with established microvascular complications, poor glycaemic control (HbA1c 7.5%), or diffuse peripheral vascular disease are still considered unsuitable for heart transplantation [26].

The original criterion for PTDM did not exclude posttransplant stress hyperglycemia, when many patients are receiving large doses of corticosteroids, which has been shown to spontaneously resolve following tapering of immunosuppressive doses [28]. It is now recommended that a diagnosis of PTDM be delayed until the recipient has been discharged from hospital and stabilized on their likely maintenance immunosuppression and in the absence of acute infection [11].

## 3. Risk Factors and Pathogenesis of PTDM

The current belief is that the pathophysiological mechanism behind PTDM differs from that of type 2 diabetes mellitus (T2DM) [29, 30]. Past studies have linked insulin resistance as a significant contributor to PTDM, thought to be attributed to mechanisms resembling those found in traditional T2DM [31]. However, there is emerging evidence to suggest that impaired insulin secretion may also be important [30]. The natural history of the diseases is similar, as the onset can be insidious and individuals may be asymptomatic for years before being clinically symptomatic [32].

**Figure 1:**
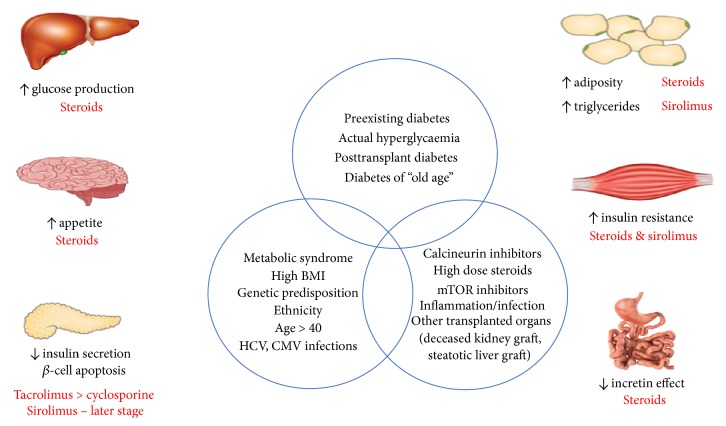
Risk factors and pathogenesis of PTDM after heart transplantation.

As illustrated in Figure 1, the risk factors for developing PTDM are largely correlated with preexisting diabetes risk, including age (>40), Body Mass Index (BMI > 25 kg/m^2^), Hepatitis C Virus (HCV) infection, African-American or Hispanic ethnicity, family history of DM, and metabolic syndrome (particularly decreased high-density lipoprotein [HDL]) [33]. However, a major factor contributing to rates of PTDM is the role of immunosuppressive agents. A meta-analysis conducted by Montori et al. [4] showed that 74% of the variability in incidence rates of PTDM can be attributed to the variation between immunosuppressive regimens, with high-dose steroids being associated with the highest incidence rates [4]. There has been a report of some reversibility of steroid-induced diabetes (“transient NODAT”) [34, 35]; however this was based on the original definition of NODAT which included the early posttransplant period.

Corticosteroids have been shown to cause hyperglycaemia through several mechanisms: induction or worsening of preexisting insulin resistance, increasing liver gluconeogenesis whilst decreasing insulin secretion, and, in the long term, by stimulating appetite and subsequent weight gain [36, 37]. In addition,* in vitro* studies have demonstrated a direct inhibitory effect of corticosteroids on insulin release by beta-cells in response to a glucose challenge and induction of beta-cell death [36]. These effects of corticosteroids are dose-dependent. A prospective randomized controlled trial that examined early corticosteroid withdrawal versus maintenance on low-dose prednisone for 6 months to 5 years following kidney transplantation failed to show a significant impact on the incidence of PTDM, suggesting that, at very low doses, the aforementioned effects of corticosteroids on insulin sensitivity and glucoregulation do not occur [38].

Calcineurin inhibitors (CNIs) (cyclosporine and tacrolimus) are critical components of most heart transplant immunosuppressive regimens, and whilst being associated with both hyperglycemia and hyperlipidemia, their effect in clinical trials is hard to interpret, due to accompanying steroid administration [39]. Whilst both drugs are believed to act similarly (via decreasing insulin secretion), tacrolimus causes a larger degree of glucose metabolism impairment than cyclosporine [39].

Mammalian target of rapamycin (mTOR) inhibitors (sirolimus and everolimus) has also been implicated in the pathogenesis of PTDM, with sirolimus being shown to increase the risk of PTDM following kidney transplantation [40, 41]. A series of case reports suggest that switching from cyclosporine to sirolimus can result in improved glycemic control [42]. However, a meta-analysis performed by Murakami et al. [43] showed conversion from CNI to mTOR inhibitor in low-to-moderate risk kidney transplant recipients actually resulted in a nonsignificant trend towards increased PTDM risk. Sirolimus has been shown to cause dose-dependent hyperglycaemia and short-term insulin resistance [44, 45]. Everolimus is a newer mTOR inhibitor and whilst there are fewer studies evaluating its effect, it is believed to increase PTDM risk similar to sirolimus [43].

The antiproliferative agents, mycophenolate mofetil (MMF) and azathioprine, have not been shown to affect glucose metabolism or insulin action and do not appear to play a significant role in PTDM.

Statins have become an integral component of the pharmacological management of heart transplant patients, due to their ability to reduce mortality [46]. A recent meta-analysis by Vallakati et al. [47] suggested that statins not only improve survival but may prevent fatal rejection episodes, decrease terminal cancer risk, and reduce the incidence of coronary vasculopathy. Whilst no studies have specifically examined the diabetogenic effect of statins amongst the post-heart transplant population, statins have been linked with a slightly increased risk of diabetes development amongst the general population, particularly amongst postmenopausal women [48]. It would be reasonable to extrapolate this risk to the transplant population; however the increased risk arising from statin therapy appears low in absolute terms.

Ultimately it is recommended that the immunosuppressive regimen be determined based solely on the best outcome for overall patient and graft survival, irrespective of PTDM risk [11].

## 4. Management of Hyperglycaemia in the Peritransplant Setting

Following solid-organ transplantation, there is a high incidence of “stress hyperglycemia” in the immediate posttransplant period [49, 50]. However, there is a notable lack of evidence in relation to the management strategies for posttransplant hyperglycemia and subsequent outcomes. The most recent guidelines from the International Society of Heart and Lung Transplantation recommend aggressive management of hyperglycemia for the duration of hospitalization, with a continuous infusion insulin regimen to be used to maintain BG below 200 mg/dL [11 mmol/L] during the intensive care unit (ICU) stay [5]. The purported rationale is to reduce the stress on beta-cells during the peritransplant period to improve their long-term function [51]. However, specific glycemic targets have not been established for patients in the immediate heart transplant setting; thus methods vary amongst transplant centres.

Currently only one prospective randomized trial examining glycaemic control following solid-organ transplant in relation to graft outcomes exists. Hermayer et al. [52] examined intensive glycaemic control (blood glucose target of 70–110 mg/dL [3.9–6.1 mM]) compared to standard-of-care (blood glucose target < 180 mg/dL [10 mM]), in the 72 hours after transplant amongst kidney transplant recipients. There was no difference for delayed graft function (*P* = 0.46). However, unexpectedly, those treated with intensive glycemic control were at greater risk of a rejection episode (*P* = 0.012), with a trend to more hypoglycemic events (*P* = 0.08). However, it is important to note this study only involved patients with established pretransplant diabetes [52].

In the setting of heart transplantation, one retrospective study demonstrated that intravenous (IV) and subcutaneous (SQ) insulin protocols with a glucose target of 80–110 mg/dL [4.5–6.1 mM] could safely be implemented in both patients with and without pretransplant diabetes [53]. However, the long-term effect of this intensive glycemic control was not studied.

In a small randomized controlled trial of 50 renal transplant recipients, Hecking et al. [51] reported that early basal insulin used to treat posttransplant hyperglycemia (<3 weeks) significantly decreased the odds of developing PTDM within the first year by 73%. A larger randomized controlled clinical trial (ITP-NODAT, clinicaltrials.org: NCT01683331) is nearing completion and has been undertaken to evaluate whether the findings of Hecking et al. are reproducible in a large, multicentre trial.

## 5. Management of Posttransplant Diabetes Mellitus

Due to the adverse impact of PTDM on posttransplant outcomes, it is important to manage the disease effectively, as poor glycaemic control has been shown to increase mortality and morbidity [14, 54–56].

For patients with type 1 diabetes and cystic fibrosis-related diabetes, continued insulin management will be maintained after transplant albeit with different insulin requirements according to feeding or immunosuppressive regimen. However, for patients with preexisting T2DM or PTDM patients, there are no studies which have established one oral agent as being safer or more efficacious. Consequently, PTDM is generally managed in accordance with the general guidelines set for the management of T2DM. Patients with preexisting T2DM whose glucose was well controlled before transplant can sometimes return to their pretransplant regimen upon discharge from hospital; however, alterations may be needed due to interactions with immunosuppressive drugs and/or development of renal dysfunction.

Due to the comorbidities of the posttransplant population and based on recommendations of the ADA [57], some authors have suggested a less aggressive HbA1c goal ranging between 7.5% and 8.0%, compared to the <7.0% recommendation for the general population [58]. In addition, HbA1c may not be an accurate indicator of glycaemic control in patients following heart transplant due to concurrent anaemia and renal impairment. Currently, there is no broad consensus regarding long-term glycemic targets for heart transplant recipients with PTDM. Heart transplant recipients face a theoretical risk of hypoglycemic unawareness from early cardiac denervation and increased risk of severe hypoglycaemia if the patient is treated with diabetic agents known to increase the risk of hypoglycaemia. However, with the advent of continuous glucose monitoring devices, it may be possible to safely aim for stricter glycemic targets. This is clearly an area where further research is needed.

## 6. Insulin-Thresholds for Use

In the immediate posttransplant period, insulin therapy is the only safe agent in the context of increased risk of lactic acidosis and single or multiorgan failure. Furthermore, insulin regimen and doses can easily be titrated and adjusted according to immunosuppressant doses, nutritional requirement, and renal impairment with theoretically no ceiling effect. At our centre, we additionally tailor insulin requirements to eating patterns with early education for insulin to carbohydrate ratio for meals and nutritional supplements. We also provide our patients with comprehensive education to manage and avoid potential hypoglycemic events. However, given that insulin administration requires multiple injections and blood capillary glucose testing and poses ongoing risk of hypoglycemia and weight gain, we aim to transition non-type 1 diabetes and CF-related diabetes to oral hypoglycaemic agents following stabilization and weaning of immunosuppressive doses. However, in long-term management of PTDM, if weaning of insulin is not possible, there is a paucity of data about the use of insulin pump or the combination of basal insulin regimen with hypoglycemic agents to improve glycaemic control and decrease hypoglycemic events and the need for multiple daily injections in the transplant setting.

## 7. Alternative Hypoglycaemic Agents

**Figure 2:**
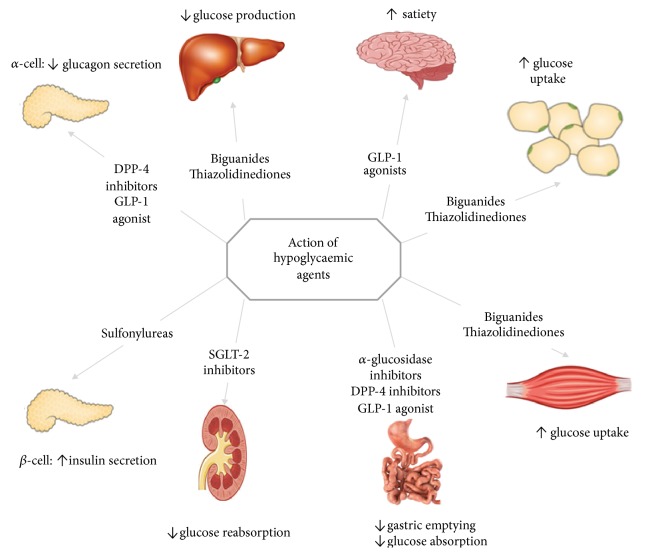
Sites of action of hypoglycaemic drugs.

An increasing variety of alternative hypoglycaemic agents are now available to treat DM in the general population. Most are orally active but some like insulin require parenteral administration.  Figure 2 illustrates their known mode of action and Table 1 summarizes their potential advantages and disadvantages in the management of PTDM after heart transplantation. Clinical experience with some of the newer classes of hypoglycaemic agents in the management of PTDM after heart transplant is minimal to nonexistent.

### 7.1. Biguanides-Metformin

Metformin is the first-line oral agent used amongst patients with T2DM amongst the nontransplant population [57]. However, its use in the management of PTDM is limited due to fears of lactic acidosis, particularly during periods of acute renal impairment or intercurrent infection. Metformin lowers glucose levels by increasing hepatic insulin sensitivity and decreasing hepatic gluconeogenesis [59]. This effect is achieved via a complex cascade, which is not fully understood and initiated by the activation of AMP-activated protein kinase [60]. Metformin is not metabolized by cytochrome 3A4 (CYP) and thus no drug-drug interactions are seen with immunosuppressive agents. There is a paucity of data relating to the use of metformin after transplantation but a single-centre retrospective study analysed its use amongst renal transplant recipients compared to thiazolidinediones [61]. Whilst it failed to show any superiority, it did demonstrate safety under close monitoring.

Metformin is renally cleared and thus is contraindicated in the setting of advanced renal dysfunction, when the estimated glomerular filtration rate (eGFR) is <30 ml/min/1.73 m^2^ [57] due to an increased risk of lactic acidosis. This is particularly relevant amongst the heart transplant population where there exists a greater prevalence of renal insufficiency [1]. A recent meta-analysis conducted by Inzucchi et al. [62] revealed that the rate of lactic acidosis with metformin use ranged from 3 to 10 per 100,000 person-years, which is indiscernible from the background rate amongst the overall diabetes population. This supports the findings of Salpeter et al. [63] which revealed no cases of fatal or nonfatal lactic acidosis across 347 trials totalling 70,490 patient years. The Food and Drug Administration (FDA) recommendations state that metformin is contraindication in men with a serum creatinine > 0.133 mmol/L and women with a serum creatinine of >0.124 mmol/L. The current guidelines for metformin dosing state that it can be used with no dose reduction with an eGFR ≥ 45 ml/min/1.73 m^2^ and a maximum dose of 1000 mg daily with an eGFR of 30–44 ml/min/1.73 m^2^ and discontinued with an eGFR < 30 ml/min/1.73 m^2^. Furthermore, metformin should be withheld at time of or before an iodinated imaging procedure and only restarted after 48 hours after the imaging procedure after reassessment of renal function especially when this was impaired at baseline.

Beyond metformin well-established diabetic effects, there has been considerable interest in its antitumour properties since an observational study published in 2005 showed a 23% decreased risk of any cancer [64]. This is particularly relevant due to the increased incidence of malignancies after cardiac transplant. However, with studies such as Mamtani et al. reporting null effects of metformin use on cancer prevention and treatment, this remains a controversial issue [65].

Based on metformin's cardiac and metabolic benefits and its potential antitumour properties, we believe that metformin is an acceptable choice for long-term management of PTDM provided eGFR > 30 ml/min/1.73 m^2^.

### 7.2. Sulfonylureas and Glinides

Sulfonylureas and glinides stimulate insulin secretion from pancreatic beta-cells, with their primary mechanism of action being to close ATP-sensitive K-channels in the beta-cell plasma membrane, initiating a sequence of events which concludes with insulin being released [66]. Due to this mechanism of action, they are prone to causing hypoglycemia, especially amongst patients with decreased GFR as they are renally cleared [67]. Sulfonylureas are one of the oldest drugs for managing diabetes, and there exists a small body of evidence to support their use for the management of PTDM [68, 69].

Whilst sulfonylureas have been shown to effectively control blood glucose levels, there remains significant controversy in relation to cardiovascular safety, beta-cell “exhaustion,” and effect on mortality, with many studies suggesting nonsignificant benefits or even increased incidence of adverse outcomes [70, 71]. Consequently, with the availability of other antidiabetic drug classes, which have stronger evidence supporting their long-term benefits, it is our recommendation that sulfonylureas not be used in the management of PTDM following heart transplantation.

### 7.3. Thiazolidinediones

Thiazolidinediones (TZDs) are oral peroxisome proliferated-activated receptor (PPAR) gamma modulators. TZDs action on PPAR-gamma increases insulin sensitivity in tissues including myocytes, adipocytes, and hepatocytes [72]. With TZD use insulin secretion is also increased, even after adjusting for the improvements in insulin sensitivity [73]. The TZDs currently available, pioglitazone and rosiglitazone, are metabolized hepatically via CYP-2C8, in contrast to the original TZD (troglitazone, now discontinued) which was metabolized via CYP-3A4 [74]. Consequently, the new generation of TZDs can be used without drug-drug interactions with the immunosuppressive agents used after transplant.

Following solid-organ transplantation, the use of TZDs for the management of PTDM has been examined in kidney and liver transplant settings, but not amongst heart transplant recipients. A series of studies have shown that TZDs either as monotherapy or in combination with another antidiabetic agent are effective at lowering HbA1c, with no significant interactions with cyclosporine or tacrolimus. However, these studies were hindered by two notable limitations. Firstly, the lack of control groups and variety in immunosuppressive regimens make it hard to comment on the efficacy of the treatment. Furthermore, they were conducted using the old criteria for NODAT diagnosis. Due to the reversible nature of PTDM diagnosed in the initial period after transplantation, when a significant number of patients were recruited for the trials, it is difficult to determine the impact of the intervention on the subsequent improvement in glucose homeostasis.

TZDs are strongly associated with an array of adverse effects, whose impact is amplified amongst the posttransplantation population. TZDs have been linked with weight gain of between 2 and 5% in monotherapy and more when used in combination with either sulfonylureas or insulin [75–77], fluid retention and heart failure [78–81], increases in fracture risk in women, but not men [82–84], and, possibly, an increased incidence of bladder cancer [85, 86]. Based on the adverse effect profile of TZDs, particularly their ability to exacerbate heart failure due to fluid retention, it is our recommendation that TZDs not be used for the management of PTDM following heart transplantation, until such a time that sufficient literature exists to show safety and noninferiority compared to other commonly used antidiabetic drugs available.

### 7.4. Incretin-Based Therapy: DPP-4 Inhibitors and GLP-1 Agonists

The incretin (INtestinal seCRETion of INsulin) system comprises two key hormones: glucagon like peptide 1 (GLP-1) and glucose-dependent insulinotropic polypeptide (GIP), which act to augment insulin biosynthesis and secretion, suppress glucagon secretion, inhibit gastric emptying, and reduce appetite [87]. Collectively, GLP-1 and GIP are known as the incretin hormones and are released from the gastrointestinal tract following glucose ingestion [88], explaining why oral glucose results in a more prominent insulin response compared to intravenous (IV) administration in healthy individuals [89]. However the insulin response to oral and IV glucose is similar in T2DM patients, indicating impairment of the incretin response [87]. In T2DM, the insulinotropic effects of GIP are trivial in comparison to GLP-1; hence, incretin system pharmacology is directed at augmenting GLP-1 [90].

Modulation of the incretin system can be used to treat diabetes, with two approved therapies currently available, dipeptidyl peptidase 4 (DPP-4) inhibitors and GLP-1 receptor agonists. The first pharmacological approach is centred around GLP-1 mimetics. GLP-1 is rapidly inactivated by the DPP-4 enzyme, with a circulating half-life of ~1.5 minutes [91], so the mimetics are required to be more resistant to DPP-4 activity than endogenous GLP-1. GLP-1 receptor agonists, such as exenatide, liraglutide, and dulaglutide, directly stimulate pancreatic beta-cells to release insulin [92]. The alternative pharmacological approach is to inhibit the DPP-4 enzyme. DPP-4 inhibitors, saxagliptin, sitagliptin, vildagliptin, alogliptin, and linagliptin, increase physiological levels of the incretin hormones [87]. Both incretin approaches have been shown to be efficacious in regard to glycaemic control and have favourable effects on weight, with GLP-1 agonists linked to weight loss, whilst DPP-4 inhibitors have been shown to have weight neutral effects [93]. Due to their glucose-dependent mechanism of action, incretin-based agents have a particularly low hypoglycemia risk [94], with severe hypoglycemic events extremely rare [95]. This lack of hypoglycemic potential is a major advantage of incretin therapy when differentiating between agents for the treatment of PTDM.

Regarding the use of GLP-1 agonists for PTDM management following solid-organ transplantation, only two case series examining liraglutide therapy in kidney [96] and pancreas [97] transplantation exist, totalling 11 patients. Whilst neither demonstrated adverse outcomes, it is difficult to comment on their suitability due to the paucity of data. Nonetheless, the recent report of a significant reduction in cardiac events and all-cause mortality with liraglutide in patients with T2DM suggests that GLP-1 agonists warrant further evaluation in the management of PTDM particularly after heart transplantation [23]. DPP-4 inhibitors, vildagliptin and sitagliptin, have been shown to be safe and efficacious for the management of PTDM following both renal [98, 99] and heart [100] transplantation and do not display drug-drug interactions with the immunosuppressive drugs used after transplant [101]. Saxagliptin is metabolized by the CYP 3A4/5 pathways [102] and hence drug-drug interactions with immunosuppressive agents will theoretically be seen, making it not suitable in the setting of PTDM.

Another favourable characteristic of incretin-based agents is their ability to be safely used in patients with impaired renal function. DPP-4 inhibitor linagliptin can be prescribed without dose reduction in renal impairment [103], whilst saxagliptin, vildagliptin, and sitagliptin can be used even in severe renal impairment, following dose reduction [104, 105]. GLP-1 agonists liraglutide and exenatide are also suitable for use in patients with mild to moderate renal impairment [106, 107].

There has been controversy regarding incretin therapy and the risk of both acute pancreatitis and pancreatic cancer, following a series of case reports which indicated a relationship. A systematic review [108] and FDA/European Medicines Agency (EMA) regulatory study [109] concluded there was no increased risk of acute pancreatitis related to incretin therapy, but they have called for further investigation. A large case-control study showed that metformin had the same risk of acute pancreatitis despite a completely unrelated mechanism of action, suggesting an underlying cause arising from diabetes pathology as opposed to a drug-specific issue [110]. There remains unanswered questions regarding pancreatic and, to a lesser degree, thyroid cancer risk arising from incretin therapy; however there is a lack of adequate long-term data, especially amongst immunosuppressed patients [111, 112].

GLP-1 agonists have been shown to have more adverse effects, specifically gastrointestinal (GI) disturbances and nausea, than DPP-4 inhibitors [113]. Given the prevalence of GI disturbances related to the immunosuppressive regimens used [114], DPP-4 inhibitors appear better suited for the management of PTDM. Another disadvantage of GLP-1 agonists is that they require subcutaneous administration whereas DPP-4 inhibitors are orally active.

DPP-4 inhibitors have been shown in animal models to exert pleiotropic effects in relation to myocardial remodelling and cytoprotection [115], which is particularly important amongst patients with PTDM, a cohort who have a significantly raised cardiovascular mortality risk [116]. The use of saxagliptin (SAVOR-TIMI 53 trial) [22], alogliptin (EXAMINE trial) [117], and sitagliptin (TECOS trial) [20] did not find a significant effect of DPP-4 inhibition on cardiovascular outcomes; however saxagliptin administration in the SAVOR-TIMI 53 Trial was associated with a significant 27% increase in hospitalizations for heart failure. Subsequent post hoc analyses of the EXAMINE and TECOS Trials reported no increase in the risk of heart failure with alogliptin or sitagliptin suggesting that the increase in heart failure observed with saxagliptin may be specific to this drug rather than a class effect [21]. Despite neutral results in the TECOS outcome study, the FDA has issued a warning about prescribing sitagliptin to patients at increased risk of heart failure. Based on the initial studies of DPP-4 inhibitors and the current FDA recommendations, sitagliptin and saxagliptin should not be used in the management of PTDM after heart transplantation. An ongoing Phase III trial to examine the cardiovascular safety of linagliptin (CAROLINA) should help provide further insight into the cardiovascular safety of DPP-4 inhibitors in humans.

There is a lack of clinical data to make a definitive recommendation regarding which incretin-based therapy is superior for the treatment of PTDM after heart transplantation, especially in relation to morbidity and mortality. The more favourable side-effect profile and ease of administration of DPP-4 inhibitors compared with GLP-1 agonists suggest that DPP-4 inhibitors will be better tolerated; however, the emerging evidence of superior cardiovascular outcomes with the use of GLP-1 agonists in T2DM indicates that larger-scale trials of both DPP-4 inhibitors and GLP-1 agonists in the treatment of PTDM are warranted.

### 7.5. SGLT-2 Inhibitors

Sodium-glucose cotransporter 2 (SGLT-2) inhibitors act via inhibition of SGLT-2 channels, which are located almost exclusively in the renal tubules and are responsible for approximately 90% of renal glucose reabsorption [118]. Inhibition of SGLT-2 reduces renal glucose reabsorption, leading to increased glucose excretion in the urine, resulting in lowering of plasma glucose levels through a mechanism which is independent of both B-cell function and insulin sensitivity [119]. Consequently, SGLT-2 inhibitors do not cause hypoglycemia.

The* EMPA-REG* outcome study was designed to test the noninferiority of empagliflozin versus placebo in relation to cardiovascular safety. The outcome of 7,020 T2DM individuals demonstrated significant reductions in major adverse cardiovascular events (*P* < 0.001), all-cause mortality (*P* < 0.001), and hospitalizations for heart failure in the empagliflozin-treated subjects (*P* = 0.002) [25]. This is significant as it was the first study of an antidiabetic drug shown to reduce the risk of death, CV death, and heart failure amongst patients with T2DM patients [120]. The* CANVAS* study explored canagliflozin in T2DM individuals at high cardiovascular risk, and its results were supportive of the EMPA-REG outcomes. Across 10,142 participants, canagliflozin-treated subjects demonstrated a 14% reduction in the composite of death from cardiovascular causes, nonfatal myocardial infarction, or nonfatal stroke (*P* < 0.001 for noninferiority; *P* = 0.02 for superiority); however this is different from the EMPA-REG study where the reduction in MACE was driven by 38% reduction in CV death.

The CANVAS study further solidified the benefits of SGLT-2 inhibitors as a class of drugs of reduced hospitalizations for heart failure by 33% (95% confidence interval, 0.52–0.87) which was similar to EMPA-REG (35% reduction) and benefits with renal outcomes [121]. These renoprotective effects are another favourable characteristic of this class of hypoglycaemic agents given the high rates of renal dysfunction in heart transplant recipients. The* EMPA-REG* study found slower progression of kidney disease and lower rates of clinically relevant renal events when empagliflozin was added to standard care [122]. Across numerous renal outcome measures, there was a significant difference in the empagliflozin group: incident or worsening nephropathy (hazard ratio 0.61; 95% confidence interval, 0.53–0.70; *P* < 0.001), progression to macroalbuminuria (hazard ratio 0.62; 95% confidence interval, 0.54–0.72; *P* < 0.001), and initiation of renal-replacement therapy (hazard ratio 0.45; 95% confidence interval, 0.21–0.97; *P* = 0.04) [122]. The* CANVAS *study showed similar benefits of canagliflozin therapy and renal outcomes: progression of albuminuria (hazard ratio 0.73; 95% confidence interval, 0.67–0.79) and 40% reduction renal-replacement therapy, or renal death (hazard ratio 0.60; 95% confidence interval, 0.47–0.77) [121].

It is worth noting that patients treated with canagliflozin in the* CANVAS *study have an increased risk of amputation compared to placebo (hazard ratio 1.97), with amputations primarily at the level of the toe or metatarsal [121]. However, no such observations were made in the EMPA-REG study and the reasons behind this remain unclear. Potential explanations for the discrepant results between the two trials might be due to the study design with CANVAS being two studies put together. In the original CANVAS study, patients were followed up for 296 weeks compared to only 104 for the CANVAS-R study. Moreover, the EMPA-REG was a “secondary prevention” trial with the inclusion criteria being patients with prior CV events whereas CANVAS had one-third primary prevention patients with the rest having secondary prevention. In the next few years, results from the* DECLARE Study *(Dapagliflozin in lower CV risk patients) will be available and will provide additional evidence regarding this novel treatment class.

The prescribing recommendations for canagliflozin and empagliflozin dosage state they should be stopped if the eGFR < 45 ml/min/1.73 m^2^. However, it is worth noting that, in the* EMPA-REG* study, patients were recruited with an eGFR as low as 30 ml/min/1.73 m^2^ [25]. Dapagliflozin dosing recommendations state it should be stopped if the eGFR < 30/ml/min/1.73 m^2^. These recommendations are based on the significant reduction in the efficacy of SGLT-2 inhibitors below these eGFR thresholds.

A pharmacokinetic study did not demonstrate any meaningful interaction between canagliflozin and cyclosporine [58].

The first study examining the safety of SGLT-2 inhibitors amongst transplant patients was recently published by our group. In 19 diabetic heart transplant patients treated with empagliflozin, there was a significant reduction in body weight (*P* = 0.05) and BMI (*P* = 0.04), mean frusemide dose (*P* = 0.05), and systolic (*P* = 0.03) and diastolic (*P* = 0.03) blood pressure [123], consistent with the results published in the* EMPA-REG *outcome. There was also a nonsignificant HbA1c reduction of 0.6%. Most importantly, amongst 147 months of cumulative empagliflozin treatment, there were no serious adverse events documented including genitourinary infections or euglycemic ketoacidosis [123], which were the main concern regarding the use of SGLT-2 inhibitors in the posttransplant setting [124].

Whilst there remains the need for larger safety trials, these preliminary results suggest that SGLT-2 inhibitors are suitable for use following heart transplantation. The beneficial effects of SGLT-2 inhibitors amongst the general diabetic population have been driven primarily by an unprecedented reduction in heart failure and hospitalizations for heart failure [25, 121]. Based on these remarkable findings, SGLT2 inhibitors are now undergoing trials in nondiabetic heart failure populations. Given the range of beneficial, nonglycemic effects seen with SGLT-2 therapy, there is the potential for SGLT-2 inhibitors to become a mainstay of PTDM management in the future.

## 8. Summary and Conclusion

Posttransplant diabetes mellitus is common after heart transplantation and is associated with increased morbidity and mortality. Despite the large number of hypoglycaemic agents currently available to treat diabetes mellitus, there is currently very little published clinical data to guide the clinician regarding the risks and benefits of individual agents in the posttransplant setting. There are serious safety concerns regarding the use of older oral agents including metformin, sulfonylureas, and thiazolidinediones in the management of PTDM after heart transplantation. Whilst the limited clinical experience with newer classes such as incretins and SGLT2 inhibitors suggests that they may have a favourable risk/benefit ratio, there is an urgent need for larger randomized controlled trials of these drugs in the management of PTDM after heart transplantation.

## Figures and Tables

**Box 1:**
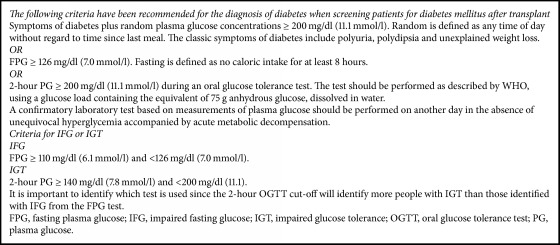
WHO and ADA criteria for the diagnosis of diabetes mellitus, impaired fasting glucose (IFG), and impaired glucose tolerance (IGT) [16, 17].

**Table 1:** Mode of action, advantages, and disadvantages of hypoglycaemic drugs.

Class	Main physiological actions	Advantages	Disadvantages
Biguanides (metformin)	↓ hepatic glucose production ↑ insulin sensitivity	Weight neutral CVD events (UKPDS [18]) No hypoglycaemia Low cost	Gastrointestinal side-effects (diarrhoea, bloating) Vitamin B12 deficiency Lactic acidosis Contraindicated in CKD, hypoxia, infections, contrast media

Sulphonylureas (glibenclamide, glipizide, gliclazide, glimepiride)	↑ insulin secretion	Microvascular benefits (UKPDS [18]) Low cost	Hypoglycaemia Weight gain Accumulation in renal impairment ? decrease ischaemic preconditioning ? QT abnormalities

Thiazolidinediones (pioglitazone, rosiglitazone)	↑ insulin sensitivity	No hypoglycaemia Sustained control ↓ triglycerides (pioglitazones) ↓ HDL-C	Oedema/heart failure Fragility fractures ↑ weight ↑ LCL-C (rosiglitazone) ? MI (meta-analysis, rosiglitazone)

*α*-glucosidase inhibitors (acarbose)	↓ carbohydrate absorption	No hypoglycaemia ↓ postprandial hyperglycaemia ?↓ CVD events (STOP-NIDDM [19]) Not absorbed systemic	Gastrointestinal side-effects Modest glycaemic benefit Frequent dosing with meals

DPP-4 inhibitors (sitagliptin, vildagliptin, saxagliptin, linagliptin, alogliptin)	↑ insulin secretion ↓ glucagon secretion	No hypoglycaemia Weight neutral Dose-adjusted in renal impairment (Linagliptin, metabolised in liver) Safe in cardiovascular disease (sitagliptin- TECOS [20], alogliptin- EXAMINE [21])	Angioedema/urticaria Arthralgia ? pancreatitis ? heart failure (saxagliptin, SAVOR-TIMI 53 [22])

GLP-1 agonists (exenatide, exenatide extended release, liraglutide, albiglutide, lixisenatide, dulaglutide, semaglutide)	↑ insulin secretion ↓ glucagon secretion ↑ satiety ↑ gastric emptying	No hypoglycaemia ↓ weight CVD benefits (LEADER [23], SUSTAIN 6 [24]) ↓ postprandial hyperglycaemia (short-acting GLP-1 agonist) ↓ fasting glucose (long acting GLP-1 agonist)	Injectable Education about administration ↑ heart rate Gastrointestinal side-effects ? pancreatitis risk C-cell hyperplasia/medullary thyroid cancer in animals

SGLT-2 inhibitors (canagliflozin, dapagliflozin, empagliflozin)	↓ renal glucose reabsorption	No hypoglycaemia Diuresis ↓ blood pressure ↓ weight ↓ CVD events (EMPA REG [25])	Genitourinary tracts infections Dehydration (dose adjustment of diuretics) Euglycaemic diabetic ketoacidosis ↑ LDL-C ? fragility fractures (canagliflozin)

Insulin	↓ hepatic glucose production ↑ glucose uptake	Theoretically no ceiling effect ↓ microvascular risk	Injectable Education about administration Hypoglycaemia Weight gain

CKD: chronic kidney disease, CVD: cardiovascular disease, and MI: myocardial infarction.

## Abbreviations

AACE: American Association of Clinical Endocrinologists
ADA: American Diabetes Association
BG: Blood glucose
BMI: Body mass index
CKD: Chronic kidney disease
CNIs: Calcineurin inhibitors
CVD: Cardiovascular disease
CYP: Cytochrome
DM: Diabetes mellitus
DPP 4: Dipeptidyl peptidase 4
eGFR: Estimated glomerular filtration rate
FDA: Food and Drug Administration
FPG: Fasting plasma glucose
GIP: Glucose-dependent insulinotropic polypeptide
GLP-1: Glucagon like peptide 1
HbA1c: Hemoglobin A1c
HDL-C: High-density lipoprotein cholesterol
ICU: Intensive care unit
IFG: Impaired fasting glucose
IGT: Impaired glucose tolerance
Incretin: Intestinal secretion of insulin
ISHLT: International Society of Heart and Lung Transplantation
IV: Intravenous
LDL-C: Low-density lipoprotein cholesterol
MI: Myocardial infarction
MMF: Mycophenolate mofetil
mTOR: Mammalian target of rapamycin
NODAT: New onset diabetes after transplant
OGTT: Oral glucose tolerance test
PG: Plasma glucose
PPAR: Peroxisome proliferated-activated receptor
Pre-Tx DM: Pretransplant diabetes mellitus
PTDM: Posttransplant diabetes mellitus
SGLT-2: Sodium-glucose cotransporter 2
T2DM: Type 2 diabetes mellitus
TZDs: Thiazolidinediones
WHO: World Health Organization.
